# Editorial: Biomarkers to evaluate rare diseases

**DOI:** 10.3389/fmmed.2023.1237089

**Published:** 2023-06-26

**Authors:** Bridget E. Bax, Dario Pacitti

**Affiliations:** ^1^ Molecular and Clinical Sciences, St George’s University of London, London, United Kingdom; ^2^ Cell Type Development, Bit.bio, Cambridge, United Kingdom

**Keywords:** rare diseases, biomarkers, castleman disease (CD), autoimmune cholestatic liver diseases, moyamoya disease (MD), neuromyelitis optica spectrum disorder (NMOsd)

There are over 7,000 distinct types of rare diseases which collectively affect a wide spectrum of body systems. Most rare disease patients experience misdiagnoses and diagnostic delays due to a poor understanding of the disease drivers. Drug development is impeded by a limited availability of natural history data, disease heterogeneity and small patient numbers. Consequently, access to effective therapies is a significant unmet need for this patient group, with only around 5% of rare diseases having regulatory approved treatments. Biomarkers are defined as characteristics that can be objectively measured and evaluated as indicators of normal biological or pathogenic processes, or pharmacological responses to therapeutic interventions. Biomarker discovery and their application thus have the potential to substantially change the trajectory of rare diseases by improving/accelerating diagnosis and supporting the development of disease-modifying therapies.

This Research Topic in Rare Diseases provides a platform for original research articles and state-of-the-art reviews that report on studies that investigate the use of predictive, diagnosis and prognostic biomarkers for rare diseases. The Research Topic includes one case report, one review and two original research articles.

The first article by Singh et al. presents an interesting index case report of a patient with Castleman disease. This rare lymphoproliferative disorder has two distinct clinical subtypes; unicentric Castleman disease (UCD) and multicentric Castleman disease (MCD), indicating single and multiple sites of lymphadenopathy, respectively. The characteristic morphologic changes of UCD, include occasional cases of overgrowth of spindled stromal and follicular dendritic cells. Although the nature and origin of these spindle cells remain elusive, some reports suggest that the cells are clonally neoplastic and may be of fibroblastic reticular cell or follicular dendritic cell. Additionally, although specific histomorphological features may aid diagnosis, there are no specific biomarkers to differentiate a reactive process mimicking UCD from true UCD. This index case reports a morphology consistent with the hyaline vascular subtype of UCD with concomitant atypical smooth muscle actin (SMA)-positive stromal spindle cell proliferation and upregulation of p53 expression. The analysis of 21 additional cases of UCD also demonstrated an increased p53 expression and SMA positive stromal cells predominantly within the paracortical and intrafollicular areas, thus strengthening the hypothesis of the stromal cellular derivation and origins of UCD. Further support for this hypothesis is provided by the increased expression of p53 within stromal cells; this also indicating its potential as a biomarker for distinguishing neoplastic and reactive processes.

The second article by Nguyen et al. provides a comprehensive review on biomarkers for the evaluation of autoimmune cholestatic liver diseases. The two categories of this group of diseases are Primary Biliary Cholangitis (PBC) and Primary Sclerosing Cholangitis (PSC). Both conditions result in hepatic bile flow impairment, leading to chronic liver injury, liver fibrosis and ultimately end stage cirrhosis. Patients with overlapping features with autoimmune hepatitis (AIH) are described to have either PBC-AIH or PSC-AIH overlap syndrome (OS). Although PBC has reliable and specific diagnostic serum autoantibody markers, PSC and OS lack such biomarkers, with diagnosis relying on imaging and invasive liver biopsies. An emerging area of biomarker investigation for both PBC and PSC is the gastrointestinal microbiome, although it is unclear whether the microbiome is a biomarker of liver disease state or a driver of disease pathogenesis. For all disease categories, unmet needs include an unclear understanding of the underlying pathogenesis, no definitive cures, and no biomarkers to prognosticate disease progression and identify complications stemming from fibrosis. The authors conclude that a combination of patient variables and different serum biomarkers (including autoantibodies) and microbiome signatures is likely to guide the care of this patient population.

The original article of Cao et al. reports the use of bioinformatic analyses to identify immune infiltration characteristics and new immunological diagnostic biomarkers in the cerebrovascular tissue of Moyamoya disease (MMD). This epidemiologically rare disorder is characterised by progressive occlusion of the terminal portion of the internal carotid artery and an abnormal vessel network at the base of the brain. Although the pathogenesis of cerebral vascular disease is unclear, evidence suggests that genetic factors play a role in disease progression. The combination and normalization of two microarray data sets dataset from the of Gene Expression Omnibus database, resulted in 348 differentially expressed genes between MMD and control groups. Following a bioinformatic workflow, BTK, FGR, SYK, and PTPN11 were shown to distinguish MMD from control patients, with the authors concluding their utility as candidate immune diagnostic biomarkers of MMD. CIBERSORT, a bioinformatics tool for characterizing cell composition of complex tissues from gene expression profiles, revealed a higher proportion of eosinophils in the specific immune infiltration landscape of MMD. These findings may provide insight for developing novel therapies.

The original research article of Bennett et al. reports a study protocol that will enable the evaluation of potential imaging and molecular biomarkers. The study, named SAkuraBONSAI, is open-label, multicenter, international and a Phase 4, and will enrol adult patients with water channel aquaporin-4 AQP4-IgG-seropositive Neuromyelitis optica spectrum disorder (NMOSD) receiving treatment with satralizumabthe. This rare autoimmune neurological disorder produces inflammatory lesions in the optic nerve, spinal cord, brainstem, and cerebrum. Some of the pro-inflammatory mechanisms are believed to be mediated in part by interleukin (IL)-6 and include an increased production of serum immunoglobulin G (IgG) autoantibodies that target the AQP4. Satralizumab is an approved, humanized, IgG2 subclass, antibody that targets the soluble and membrane-bound form of the IL-6 receptor. Anti-CD20 agents such as rituximab (RTX) are commonly used as maintenance therapies in NMOSD, but some individuals remain inadequately controlled despite treatment. The SAkuraBONSAI study aims to advance the understanding of NMOSD disease progression and treatment response in patients who are newly diagnosed and treatment-naïve, and in individuals who have responded inadequately to RTX. The incorporation of comprehensive imaging, fluid biomarker, and clinical assessments into the study design will enable new insights into the mechanism of action of satralizumab, and provide the opportunity to evaluate clinically relevant neurological, immunological, and imaging markers.

We hope these articles will lead to new avenues of investigation, and ultimately improved diagnoses and the availability of disease-modifying therapies.

